# Early Periprosthetic Metastasis Following Total Hip Replacement in a Patient With Breast Carcinoma: A Case Report and Review of Literature

**DOI:** 10.4021/jocmr618w

**Published:** 2011-07-26

**Authors:** Ravi Badge, Hiren Divecha, David Sochart

**Affiliations:** a North Manchester General Hospital, Delaunays Road, Manchester M8 5RB, UK

## Abstract

Early periprosthetic osteolysis following total hip replacement (THR) as a result of septic etiology has been well understood. Periprosthetic bone loss as a result of metastatic infiltration is an uncommon and infrequent cause of early, progressive loosening of joint replacement prosthesis. Proximal femur has been the most common site of involvement compared to acetabular prosthesis. The rarity of this clinical entity can lead to delay in definitive diagnosis and management, thus affecting the final outcome. Breast is the commonest site of carcinoma in female patients despite which not many cases of periprosthetic metastasis have been reported in the literature. We present the first case of extensive, isolated periacetabular bone destruction following a THR in a 59 years old female patient with a history of breast carcinoma. Patients with known primary malignancy should be screened thoroughly before operation and should be followed regularly after joint replacement surgery to detect any metastatic foci around the prosthesis.

**Keywords:**

Periprosthetic metastasis; Total hip replacement; Breast carcinoma

## Introduction

Total hip replacement (THR) is one of the most successful and cost-effective operations in restoring function and mobility to patients with arthritis. Aseptic and septic loosening are significant causes of its failure over time, but with the use of antibiotic prophylaxis and improvements in the bio-mechanical properties and fixation of implants, the longevity of THR has increased [1]. Periprosthetic bone loss due to metastatic infiltration is uncommon but an important clinical entity, and can contribute to early periprosthetic loosening and failure.

Patients presenting with pain after a joint replacement are usually investigated to rule out any septic or aseptic etiology and thus rare occurrence of metastatic infiltration get overlooked and this may delay the appropriate management in such situations. Therefore it is essential to consider the possibility of metastatic involvement of the bone following joint replacement as one of the differential diagnosis and should be promptly investigated with the help of the multidisciplinary team.

Metastatic infiltration around a THR is more commonly reported around femoral prosthesis compared to its acetabular counterpart. There has been only one case of acetabular involvement in a patient with gastric carcinoma which has been reported in English literature [2]. Although breast is the most common site of primary in female patients there is only one case report so far in the non-English literature with involvement of femoral component [3]. We present the first case of early, extensive periacetabular bone loss following THR as a result of metastatic infiltration in a patient with a history of breast carcinoma.

## Case Reports

**Figure 1.**
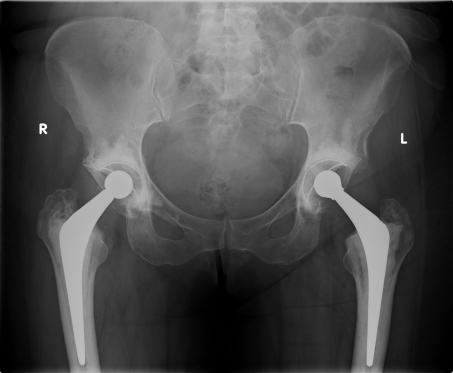
X-ray pelvis following total hip replacement.

A 59 years old female patient presented with bilateral hip osteoarthritis and underwent staged bilateral cemented THRs (right followed by left). The left hip replacement was performed in August 2008 (Fig. 1). Intra-operative findings during both hip replacements were suggestive of osteoarthritis with no signs of metastasis. The patient made an uneventful recovery after both procedures and was mobilizing well without any discomfort.

This patient had a history of a grade III invasive ductal carcinoma (known to carry a favorable prognosis) of the right breast which had been treated with lumpectomy and radiotherapy in July 2007. The patient was under regular review by the oncologists and was subsequently given the all clear.

**Figure 2.**
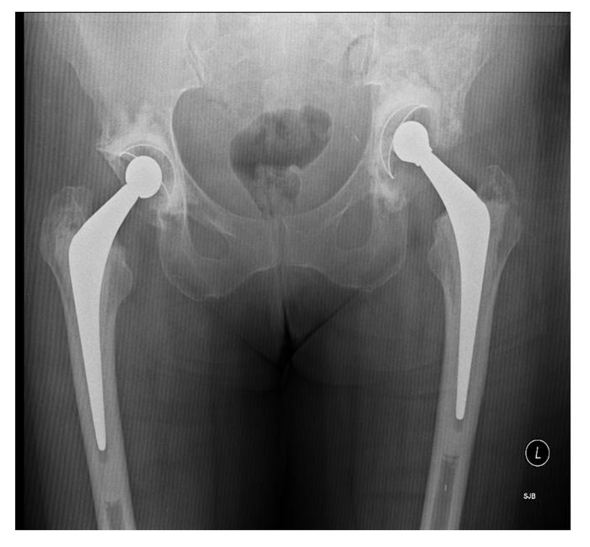
Eight month follow-up X-ray showing isolated periacetabular bone loss in left hip.

Eight months after left THR, the patient was referred by her family doctor with increasing pain and limping arising from left hip. After thorough clinical assessment, the patient underwent X-ray of the pelvis and baseline blood investigations along with C- reactive protein (CRP) and erythrocyte segmentation rate (ESR). The x-ray revealed extensive destruction of periacetabular bone in the left hemipelvis (Fig. 2). The blood investigations were essentially normal apart from a CRP of 22 and alkaline phosphatase increased to 432 (normal range 30-150)

**Figure 3.**
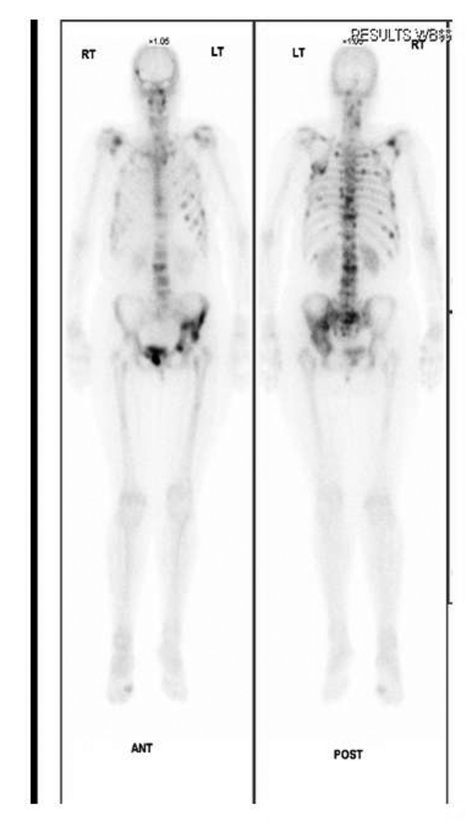
Radinucleotide bone scan confirms increased uptake in left hemipelvis.

**Figure 4.**
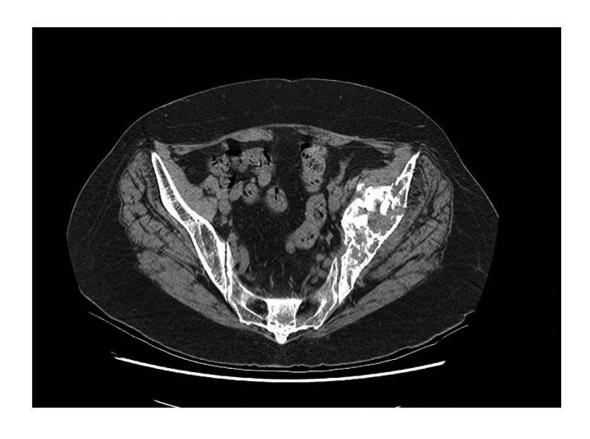
CT scan showing periacetabular bone loss.

**Figure 5.**
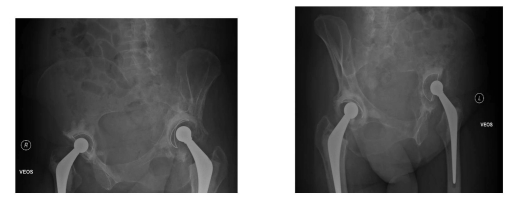
Judet view revealing rapid progression of osteolysis and migration of left acetabular component.

An urgent isotope bone scan and computed tomography (CT) of her pelvis were performed. The three phase bone scan demonstrated intense uptake in the left acetabulum, superior pubic ramus and iliac crest region confirming the presence of metastasis. It also revealed widespread bony metastasis involving the axial skeletal as well as head and neck region (Fig. 3). The CT scan of the pelvis confirmed extensive destruction of the left iliac wing (Fig. 4). X-rays of pelvis taken few months after initial diagnosis showed rapid progression of the periacetabular bone loss and superior migration of the acetabular component. The femoral prosthesis did not show any evidence of osteolysis (Fig. 5).

The patient was referred back to her oncology team for further assessment and management. The widespread metastases were confirmed to be secondary to her breast carcinoma and treated with palliative radiotherapy and hormone therapy.

**Table 1:** Review of Reported Cases of Metastatic Infiltration Around Hip Replacement From English Language Literature

Case report	Primary	Site of involvement	Duration since hip replacement	Treatment	Survival after detection
Dramis et al. 2008	Renal Cell Carcinoma	Proximal Femur	6 months	Palliative	6 months
O'Shea et al. 2006	Lung Adenocarcinoma	Proximal Femur	60 months	Revision usncemented THR	6 weeks
	Malignant Immunoblastic Lymphoma	Proximal Femur	156 months	Excision arthroplasty	20 weeks
	Gastric Carcinoma	Acetabulum	4 months	Girdlestone resection arthroplasty	10 weeks
Allain et al. 1998	Lung Squamous cell carcinoma	Synovium around hip	60 months	Revision THR	3 months
Schmidt et al. 1996	Ovarian Carcinoma	Proximal femur	21 months	Palliative	10 months
	Hepatocellular Carcinoma	Proximal femur	52 months	Revision THR	Not available
Donohoe et al. 1987	Non-Hodgkin's Lymphoma	Proximal femur	Not Available	Not available	Not available
Kim et al. 1986	Lung Adenocarcinoma	Proximal femur	8 months	Hip disarticulation	11 months

## Discussion

Infection, aseptic loosening and ischio-pubic osteolysis are the well documented causes of periprosthetic bone loss after joint replacement surgery [1, 3]. Metastatic infiltration around a joint replacement is uncommon but has been an emerging concern in last two decades as the survival of patients following malignancy and longevity of joint replacements has been increasing. Periprosthetic osteolysis can result in massive bone loss around the implant, leading to loosening of the components and periprosthetic fractures.

Occurrence of primary malignant tumors around prosthetic joint replacements has been more frequently reported than metastatic infiltration [4, 5]. Thyroid, breast, lung, gastric, hepatocellular, prostate, Kidney and ovarian carcinomas and non-Hodgkin's lymphoma are the primaries identified so far leading to metastasis around hip replacement [2, 6-10].

The pathoetiology behind metastatic infiltration around orthopaedic implants has been well described. An increased vascularity due to surgical trauma and the healing process leads to a predilection of metastatic deposition around joint replacement sites [11]. In the setting of a bony metastasis, periprosthetic osteolysis may be due to direct metastatic infiltration or an inflammatory reaction to the metastatic deposit leading to necrosis of bone and thus loosening of component [5].

Periprosthetic osteolysis due to metastatic infiltration is reported more commonly around the femoral component than the acetabulum. Breast is the commonest site of carcinoma in female patients and despite which not many cases of periprosthetic metastatic involvement following hip replacement have been reported in literature. O’Shea et al. reported the first case of isolated periacetabular metastatic involvement following THR in a patient with gastric carcinoma [9]. To our knowledge, this is the first reported case of metastatic breast carcinoma presenting with early, isolated, extensive periacetabular bone loss following THR.

Schmidt et al. in their experience of two cases of metastatic, periprosthetic osteolysis have admitted that the delay in actual diagnostic etiology could lead to major physical and psychological trauma to patient. They recommended that apart from routine investigations, a tissue biopsy must be obtained before undertaking any revision procedure and intra-operative frozen section analysis of tissue should be obtained during the revision procedure to rule out malignancy as well as infection [7]. Importantly, it has been reported that the number of cases of periprosthetic osteolysis due to metastases has been initially incorrectly attributed to infection, thus delaying its appropriate management [4, 9, 12].

As the number of cases reported in the literature is very few and infrequent, it is difficult to draw a causal relationship between the nature of the primary tumor and periprosthetic osteolysis. The time interval between detection of periprosthetic metastasis and a hip replacement has been variable with very few reported cases diagnosed within a year [2, 6-10]. Patient survival after detection of metastases around hip replacements has been reported to be poor. It is therefore necessary to get a detailed history, thorough physical examination, routine as well as tumor specific blood tests along with a radio-nucleotide bone scan and CT in patients presenting with painful joint replacements. The early detection can improve the survival and quality of life in this group of patients.

### Conclusion

Metastatic infiltration along with septic and aseptic loosening should be considered as a potential cause of early periprosthetic bone loss. Detailed history and physical examination along with radio-nucleotide bone scan, CT and recommended blood tests should form part of investigations in patient with periprosthetic bone loss. In cases with atypical presentation tissue diagnosis should be obtained either by biopsy or aspiration of the joint before revision procedure is considered.

Patients with a known history of malignancy should be screened with a pre-operative bone scan to rule out any metastatic infiltration. This group of patients should be made aware about the possibility of metastatic infiltration around joint replacement prosthesis and should have a regular follow-up at short intervals to detect any early bone loss.
